# First Principle Study on the Z-Type Characteristic Modulation of GaN/g-C_3_N_4_ Heterojunction

**DOI:** 10.3390/molecules29225355

**Published:** 2024-11-14

**Authors:** Meng-Yao Dai, Xu-Cai Zhao, Bo-Cheng Lei, Yi-Neng Huang, Li-Li Zhang, Hai Guo, Hua-Gui Wang

**Affiliations:** 1 Xinjiang Laboratory of Phase Transitions and Microstructures in Condensed Matter Physics, College of Physical Science and Technology, Yili Normal University, Yining 835000, China; dmy153097@sina.com (M.-Y.D.); zxc85619876@sina.com (X.-C.Z.); lbc0428@sina.com (B.-C.L.); ynhuang@nju.edu.cn (Y.-N.H.); suyi2046@sohu.com (H.-G.W.); 2 National Laboratory of Solid State Microstructures, School of Physics, Nanjing University, Nanjing 210093, China; 3 Department of Physics, Zhejiang Normal University, Jinhua 321004, China

**Keywords:** first principles, GaN/g-C_3_N_4_, optical properties, external electric field

## Abstract

This study investigates the stability, electronic structure, and optical properties of the GaN/g-C_3_N_4_ heterojunction using the plane wave super-soft pseudopotential method based on first principles. Additionally, an external electric field is employed to modulate the band structure and optical properties of GaN/g-C_3_N_4_. The computational results demonstrate that this heterojunction possesses a direct band gap and is classified as type II heterojunction, where the intrinsic electric field formed at the interface effectively suppresses carrier recombination. When the external electric field intensity (E) falls below −0.1 V/Å and includes −0.1 V/Å, or exceeds 0.2 V/Å, the heterojunction undergoes a transition from a type II structure to the superior Z-scheme, leading to a significant enhancement in the rate of separation of photogenerated carriers and an augmentation in its redox capability. Furthermore, the introduction of a positive electric field induces a redshift in the absorption spectrum, effectively broadening the light absorption range of the heterojunction. The aforementioned findings demonstrate that the optical properties of GaN/g-C_3_N_4_ can be precisely tuned by applying an external electric field, thereby facilitating its highly efficient utilization in the field of photocatalysis.

## 1. Introduction

Photocatalytic technology, which harnesses solar energy, has been widely applied in various domains including air purification, water splitting for hydrogen production, and self-cleaning capabilities [1,2,3]. The essence of photocatalytic technology lies in the development of efficient photocatalysts. However, the utilization efficiency of visible light by conventional photocatalysts such as TiO_2_ [4], ZnO [5], and WO_3_ [6] remains significantly low, falling below 5% [7]. Consequently, current research endeavors to enhance the light absorption range of photocatalysts in the visible spectrum while simultaneously augmenting their efficiency and stability during photocatalytic reactions [8]. Two-dimensional photocatalysts, such as g-C_3_N_4_ [9,10], TiS_3_ [11]_,_ and TiS_2_ [12], demonstrate a remarkable responsiveness to visible light and exhibit exceptional catalytic efficiency, surpassing traditional photocatalysts such as TiO_2_ [13]. Its extensive specific surface area facilitates the formation of additional reactive sites, leading to a significant enhancement in photocatalytic performance [14,15]. However, the rapid recombination of photogenerated carriers and the limited range of light absorption continue to pose significant challenges in enhancing the photocatalytic efficiency of g-C_3_N_4_ [16]. Currently, the construction of heterojunctions represents a promising strategy for enhancing the efficiency of photocatalysts. The key factor of heterojunctions as excellent photocatalysts is the formation of a built-in electric field at their interface, which effectively mitigates the rapid recombination of photogenerated carriers and optimizes the optical properties of these systems [17,18]. The hydrogen production rates of the three g-C_3_N_4_-based heterojunctions, g-C_3_N_4_/SiOC, g-C_3_N_4_/WS_2_, and g-C_3_N_4_/CeO_2_, presented in Table 1 exhibit a significant enhancement compared to that of single-layer g-C_3_N_4_, with an impressive efficiency increase ranging from 46 to 93 times. This finding strongly indicates that the photocatalytic efficiency can be greatly enhanced by constructing g-C_3_N_4_-based heterojunctions. The band gap of these three heterojunctions is smaller than that of monolayers, indicating a significant alteration in the heterojunction structure compared to single layers. Furthermore, all three heterojunctions exhibit a type II band alignment, which is observed to significantly enhance photocatalytic efficiency compared to single-layer g-C_3_N_4_.

**Figure 1:**
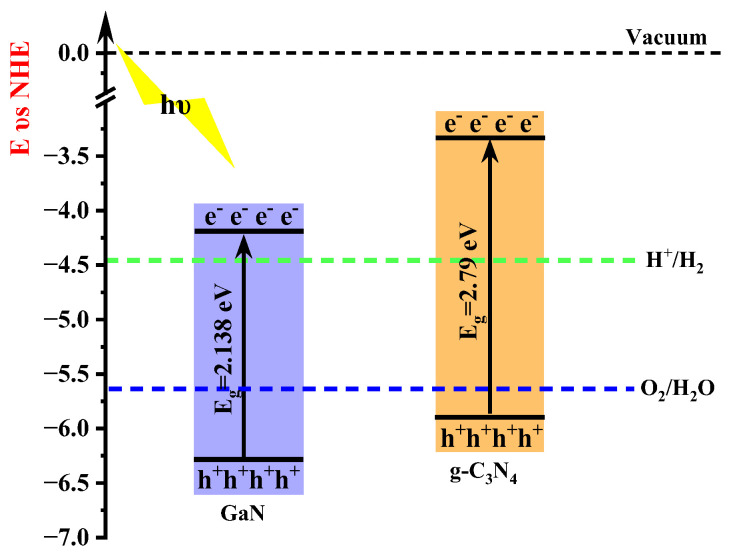
Schematic diagram illustrating the band alignment between a single layer of g-C_3_N_4_ [20] and GaN [27].

After analyzing the experimental results, it can be concluded that type II heterojunctions based on g-C_3_N_4_ exhibit superior photocatalytic efficiency compared to single-layer g-C_3_N_4_. Therefore, when selecting efficient photocatalysts, emphasis should be placed on g-C_3_N_4_-based type II heterojunctions. The band structure of the type II heterojunction is staggered, encompassing two distinct semiconductor materials, one of which must be satisfied as an oxidized type with a sufficiently low valence band position, and the other semiconductor as a reduced type with a sufficiently high conduction band position. Figure 1 illustrates the band alignment of g-C_3_N_4_ and confirms its adequately high conduction band position [23,24,25]. Accordingly, only another two-dimensional monolayer semiconductor needs to be chosen while ensuring its lower valence band position. Through screening, GaN was identified as meeting these criteria (refer to Figure 1) [26]. Based on this hypothesis, forming heterojunctions between GaN and g-C_3_N_4_ will lead to enhanced photocatalytic efficiency for type II heterojunctions.

Consequently, it is plausible to anticipate the successful realization of type II GaN/g-C_3_N_4_. In this study, we employ first principle calculations to construct GaN/g-C_3_N_4_ and investigate its electronic structure and optical properties. Our findings unveil the significant potential of this heterojunction in photocatalysis, providing theoretical support for advancing efficient and stable photocatalysts. These results contribute to the application and further development of photocatalysis technology.

However, the photocatalytic efficiency of the heterojunction remains limited due to the high probability of recombination between photogenerated electron–hole pairs and the excessive loss of photogenerated carriers [28,29]. The band edge positions of the heterojunction can be precisely tuned by applying an external electric field and carefully adjusting its intensity and direction, leading to a notable enhancement in its photocatalytic performance. The proposed approach introduces novel concepts and methodologies that make a significant contribution to the advancement of photocatalytic materials, enhancing their efficiency and stability [30,31]. The influence of an external electric field on the electronic structure of the SeZrS/SeHfS heterojunction was investigated by Yang et al. [32]. Their findings demonstrate that the application of an electric field with an intensity below −0.2 V/Å or above 0.2 V/Å induces a transition from a type I to a type II heterojunction, resulting in interlaced bands. This transition significantly facilitates the efficient separation of photogenerated carriers, thereby enhancing photocatalytic efficiency. Zhao et al. [33] utilized an external electric field to modulate the electronic structure of a PtSe_2_/ZrSe_2_ heterojunction, resulting in a tunable band gap within the range of 0–0.25 eV. The bandgap initially widens and then rapidly narrows as the external electric field shifts from negative to positive values, promoting electron transitions. Moreover, under specific ranges of electric field intensity (−0.05 V/Å < E < 0.01 V/Å and −0.17 V/Å < E < 0.22 V/Å), the heterojunction type transforms from type I to type II, leading to enhanced photocatalytic performance. The aforementioned statement underscores the capacity of an external electric field to manipulate the migration pathways of photogenerated carriers, fine-tune the band alignment of the heterojunction, and optimize the photocatalytic process. Ultimately, this enhancement in heterojunction photocatalytic efficiency confers evident advantages. Consequently, the objective of this study is to construct GaN/g-C_3_N_4_ utilizing the first principle method and to manipulate its optical properties through the introduction of an external electric field. Our aim is to provide novel perspectives and theoretical models that will advance the development of highly efficient photocatalytic materials.

## 2. Model Structures and Stability

**Figure 2:**
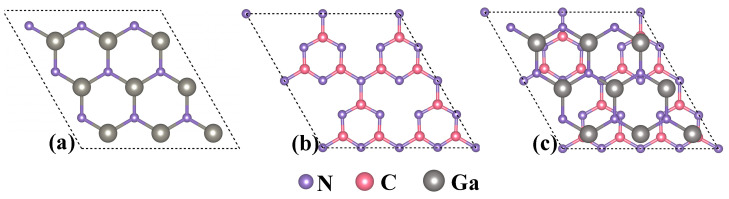
Structure model top views of (**a**) GaN supercell, (**b**) g-C_3_N_4_ supercell, and (**c**) GaN/g-C_3_N_4_ heterojunction.

The present study meticulously constructed a comprehensive model GaN/g-C_3_N_4_, comprising a total of 46 atoms (as depicted in Figure 2). First, the bulk phases of g-C_3_N_4_ and GaN were cut along the (001) plane to obtain two-dimensional g-C_3_N_4_ (a = b = 4.779 Å) and GaN (a = b = 3.210 Å). Then, based on lattice matching, the single layers of both phases were expanded to form g-C_3_N_4_ (2 × 2 × 1) and GaN (3 × 3 × 1), ultimately constructing a heterojunction with g-C_3_N_4_ as the bottom layer. To effectively mitigate the impact of interlayer coupling, a substantial vacuum layer measuring 20 Å was deliberately incorporated along the c-axis direction.

**Figure 3:**
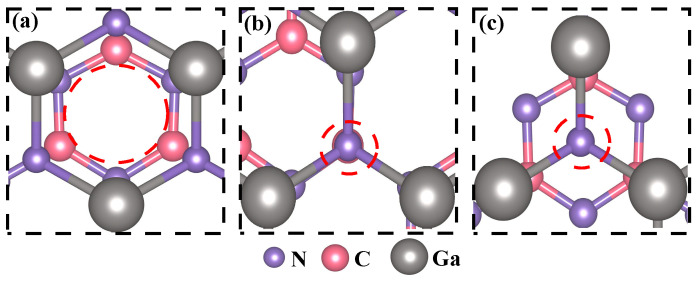
Three modes of GaN/g-C_3_N_4_ heterojunction (**a**) Model I, (**b**) Model II, (**c**) Model III. Red dashed circles represent the alignment of GaN single-layer and g-C_3_N_4_ single-layer stacks.

After conducting meticulous calculations, it was conclusively determined that the lattice mismatch rate between GaN and g-C_3_N_4_ in the heterojunction is a mere 0.9%. This result unequivocally demonstrates the feasibility of establishing a stable heterojunction between these two materials. The three GaN/g-C_3_N_4_ models depicted in Figure 3 exhibit distinct stacking patterns: Model I shows precise alignment of the N-Ga ring of the GaN layer above the N-C ring of g-C_3_N_4_, as seen in Figure 3a; Model II features accurate positioning of the N atom of the GaN layer over that of the g-C_3_N_4_ layer, as shown in Figure 3b; and Model III positions the N atom of the GaN layer at the center of the N-C ring in the g-C_3_N_4_ layer, as illustrated in Figure 3c. Additionally, we have accurately computed the total energies by employing TS and Grimme dispersion correction methods, respectively, as summarized in Table 2. The calculation results show that the total energy value calculated by the TS method is lower, thereby showing that the TS method provided a more accurate reflection of the physical nature of intermolecular dispersion. Consequently, the calculation results are based on the TS method. Analyzing the total energy across three different modes reveals that model III exhibits the lowest calculated total energy using the TS method, which indicates its superior stability. Therefore, we chose model III as the structural model for GaN/g-C_3_N_4_ in this paper.

**Figure 4:**
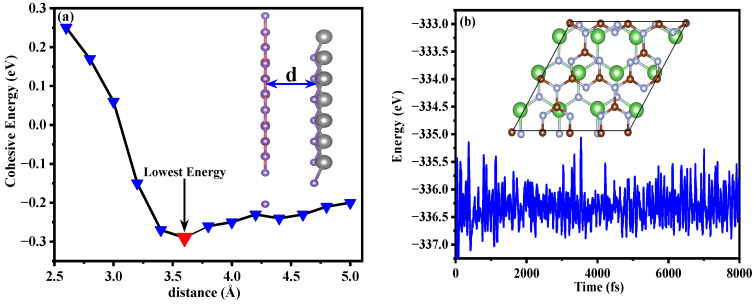
(**a**) The relationship between the binding energy of GaN/g-C_3_N_4_ and the interlayer spacing d; (**b**) The energy variation in GaN/g-C_3_N_4_ during the AIMD simulation lasting for 8 ps at 300 K, with the inset showing the top view of the final structure from the AIMD simulation. The illustrations shown in (**a**) depict gray balls representing Ga atoms, purple balls representing N atoms, and pink balls representing C atoms. The illustrations in (**b**) show green balls representing Ga atoms, gray balls representing N atoms, and brown balls representing C atoms.

To study the structural stability of GaN/g-C_3_N_4_, we calculated the binding energy at various interlayer distances. The expression for this binding energy (E_coh_) is as follows [34]: E_coh_ = E_T_(GaN/g-C_3_N_4_) − E_T_(GaN) − E_T_(g-C_3_N_4_), where the total energies of the GaN/g-C_3_N_4_, the monolayer GaN, and the monolayer g-C_3_N_4_ are denoted as E_T_(GaN/g-C_3_N_4_), E_T_(GaN), and E_T_(g-C_3_N_4_), respectively. The presence of a negative binding energy signifies the inherent stability of the heterojunction structure [35]. The heterojunction structure is considered stable when the interlayer distance reaches 3.6 Å, as depicted in Figure 4a, with a corresponding minimum binding energy of −2.960 meV/Å^2^.

To further validate the thermal stability of GaN/g-C_3_N_4_, we employed the DS-PAW (version 2023a) [36] first principle plane wave calculation software to conduct ab initio molecular dynamic (AIMD) simulations of the system at 300 K (equivalent to room temperature), as depicted in Figure 4b. After 8000 calculation steps within a timeframe of 8 ps, the system exhibits remarkable stability. The structural integrity remains intact without any chemical bond breakage, accompanied by minimal energy fluctuations. These findings underscore the exceptional thermodynamic stability of the heterojunction system at 300 K [37,38].

The lattice mismatch energy (E_miscoh_) associated with the GaN/g-C_3_N_4_ is expressed by the following equation [39]: E_miscoh_ = E(g-C_3_N_4_)_a1_ + E(GaN)_a2_ − E(g-C_3_N_4_)_a1′_ − E(GaN)_a2′_ where the total energies of the monolayer g-C_3_N_4_ and GaN supercells are denoted as E(g-C_3_N_4_)_a1_ and E(GaN)_a2_, respectively, when their lattice constants are set to a. Furthermore, E(g-C_3_N_4_)_a1′_ represents the total energy of the monolayer g-C_3_N_4_ with a lattice constant of a_1′_, while E(GaN)_a2′_ indicates the total energy of the monolayer GaN with a lattice constant of a_2′_. The lattice mismatch energy of the GaN/g-C_3_N_4_ is calculated to be −1.230 meV/Å^2^, indicating its remarkable stability. To further investigate the interaction forces at the interfaces of the heterojunction, we introduced the van der Waals energy (ΔE_vdw_) [40] as a powerful tool for analyzing this particular heterojunction. The mathematical formulation of this energy can be expressed as follows: ΔE_vdw_ = |E_coh_| + |E_miscoh_|, the absolute value of the binding energy for the heterojunction, denoted as |E_coh_|, and the absolute value of the lattice mismatch energy within the system, denoted as |E_miscoh_|, are crucial factors in this study. Notably, a van der Waals energy measurement of 4.19 meV/Å^2^ indicates that the interaction force between interfaces is primarily governed by van der Waals forces.

## 3. Analysis and Discussion

### 3.1. Electronic Structure and Optical Properties

#### 3.1.1. Energy Band Structure and Electronic Density of States

**Figure 5:**
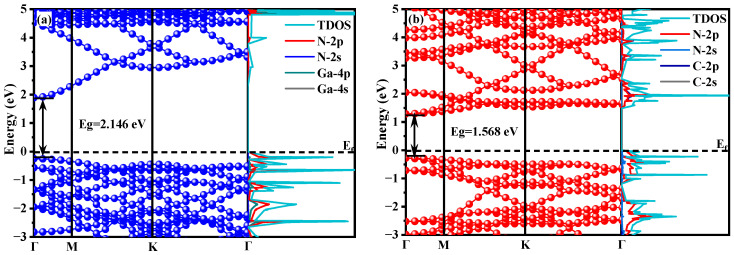
Energy band diagrams (left) and partial DOS diagrams (right) of monolayer GaN (**a**); monolayer g-C_3_N_4_ (**b**).

**Figure 6:**
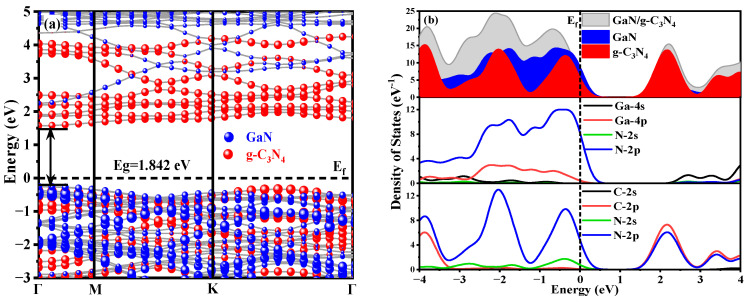
GaN/g-C_3_N_4_ heterojunction: (**a**) energy band structure diagram; (**b**) DOS diagram.

This study presents a comprehensive analysis of the band structure and electron density of states for monolayer g-C_3_N_4_, monolayer GaN, and the GaN/g-C_3_N_4_, as illustrated in Figure 5 and Figure 6. Based on the presented data, this study focuses on the energy range spanning from −3 to 5 eV, with the Fermi level serving as the reference point at 0 eV. Moreover, the conduction band minimum and valence band maximum are both located at the identical symmetry point Γ, indicating a direct electron transition in both systems. Additionally, precise calculations demonstrate that monolayer GaN possesses a bandgap width (E_g_) of 2.146 eV, which closely aligns with the calculated value of 2.138 eV by Yi et al. [27], exhibiting an error margin of only 0.37%. Conversely, monolayer g-C_3_N_4_ exhibits a narrower gap at 1.568 eV, deviating from the calculated result of 1.62 eV by Oreshonkov et al. [41] with a discrepancy of 3.2%. The electron density of state (DOS) diagram on the right provides evidence that N-2p states make up most of the valence band in monolayer GaN, accompanied by a minor contribution from Ga-4p states. Moreover, both the conduction band and a portion of the valence band in monolayer GaN are primarily influenced by N-2s states, consistent with observations made in its corresponding band structure diagram.

The band structure diagram of the GaN/g-C_3_N_4_ is depicted in Figure 6a, where the valence band maximum (VBM) and conduction band minimum (CBM) coincide at the same Γ point, indicating a direct bandgap nature with an energy value of 1.842 eV. This finding aligns consistently with previously reported literature values [42]. The CBM of GaN/g-C_3_N_4_ is predominantly determined by g-C_3_N_4_, while the VBM is primarily contributed by GaN, indicating a type-II alignment in GaN/g-C_3_N_4_. The present finding is in line with the experimental outcomes reported by Sarkar et al. [26]. Compared to monolayer systems, the two materials in GaN/g-C_3_N_4_ exhibit overlapping but maintain their respective independent electronic constructions. The combination of different materials in this process allows for the utilization of complementary advantages and the optimization of performance. Additionally, the interaction at the heterojunction interface significantly enhances the efficiency of separating photon-generated carriers.

The conduction band of the heterojunction is primarily contributed to by C-2p and N-2p states near the Fermi level, while the valence band predominantly consists of N-2p and Ga-4p states, as depicted in Figure 6b. The conduction band of the heterojunction is primarily contributed to by C-2p and N-2p states near the Fermi level, while the valence band predominantly consists of N-2p and Ga-4p states, as illustrated in Figure 6b, which can be attributed to the proximity of the valence band to the Fermi level, facilitating electron transitions from the valence band to the conduction band. Moreover, there exists an energy level overlap between the orbitals of both materials, indicating a significant hybridization of orbitals between Ga atoms and N atoms, which promotes electron transfer from Ga-4p states to N-2p states, resulting in electron accumulation at N atoms located at the heterojunction interface.

#### 3.1.2. Work Function and Effective Mass

**Figure 7:**
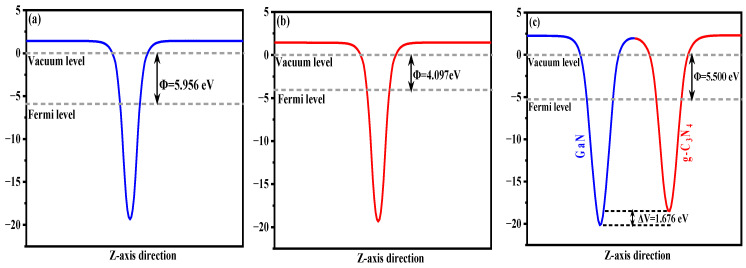
Work function diagrams of (**a**) GaN; (**b**) g-C_3_N_4_; and (**c**) GaN/g-C_3_N_4_ heterojunction (The vacuum energy level in this article is assumed to be at the reference point of 0 eV).

The work function (Φ) represents the minimum energy required for electrons to transition from the interior of a semiconductor to its surface. In this study, we conducted calculations to determine work functions for monolayer g-C_3_N_4_, monolayer GaN, and GaN/g-C_3_N_4_. The calculation formulation is outlined as follows [43]: Φ = E_vac_ − E_fer_. Here, the symbol E_vac_ denotes the vacuum energy level, while E_fer_ signifies the Fermi energy level. As depicted in Figure 7, the Φ of monolayer GaN and monolayer g-C_3_N_4_ are 5.956 eV and 4.097 eV, respectively, reaffirming the findings reported in the existing literature [44]. Compared to the GaN monolayer system, the Φ of the GaN/g-C_3_N_4_ was reduced to 5.500 eV, indicating enhanced electron excitation at the interface of this heterojunction. By comparing the minimum potential energy of GaN and g-C_3_N_4_, a potential difference of 1.676 eV between them is observed. It is deduced that an inherent electric field forms in the heterojunction, with its direction pointing from the g-C_3_N_4_ layer towards the GaN layer. Such an electric field facilitates to improve carrier mobility and efficient separation of photo-generated electron–hole pairs at the interface, ultimately resulting in a significant enhancement of photocatalytic performance for the heterojunction [45].

To gain a more comprehensive understanding of the migration behavior of electrons and holes, this study conducts an analysis by calculating the effective masses (m_e_*, m_h_*) and their ratio D = m_e_*/m_h_*, for monolayer GaN, monolayer g-C_3_N_4_, and GaN/g-C_3_N_4_. The calculation results, expressed by formulas as follows [46], are presented in Table 3:

$$m^{*}=\hbar^{2}/(\frac{\partial^{2}E}{{\partial k}^{2}})$$

Here, the *∂^2^E/∂k^2^* represents the second-order derivative of the E–K curve. The effective masses (m_e_* and m_h_*) of GaN/g-C_3_N_4_ are found to be 0.67 and 1.29, respectively; these values increase compared to those observed in the single-layer. The D value generally denotes the degree of separation between electrons and holes, effectively characterizing disparities between electrons and holes within a given system. A high D value indicates a significant degree of separation between electrons and holes within the system, resulting in an increased number of available electrons and holes for participation in photocatalytic water splitting reactions. In comparison to monolayer systems, GaN/g-C_3_N_4_ exhibits the highest D value, indicating a preference for holes and reducing the likelihood of electron–hole recombination. These findings suggest that the construction of heterojunction significantly enhances the efficiency of electron–hole separation, thus anticipating a substantial improvement in its photocatalytic performance.

#### 3.1.3. Difference in Charge Density

**Figure 8:**
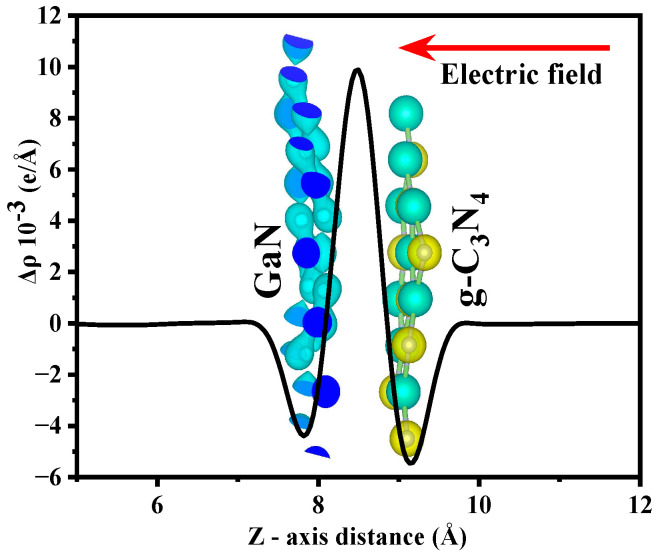
Plane-averaged differential charge density diagram of GaN/g-C_3_N_4_. The inset presents a three-dimensional diagram illustrating the differential charge density, with the yellow region indicating electron accumulation and the cyan region representing electron depletion.

In order to gain a more comprehensive understanding of the charge redistribution occurring at the heterojunction interface, this study employs formula [47] Δρ = ρ(GaN/g-C_3_N_4_) − ρ(GaN) − ρ(g-C_3_N_4_) to determine the planar-averaged differential charge density for GaN/g-C_3_N_4_. The charge densities of the GaN/g-C_3_N_4_, monolayer GaN, and monolayer g-C_3_N_4_, respectively, represent ρ(GaN/g-C_3_N_4_), ρ(GaN) and ρ(g-C_3_N_4_). As illustrated in Figure 8, a depletion of charge occurs in the GaN layer while a corresponding accumulation takes place in the g-C_3_N_4_ layer at the interface of GaN/g-C_3_N_4_. This migration of charges from the GaN layer to the g-C_3_N_4_ layer generates an internal electric field that is directed from the g-C_3_N_4_ towards the GaN region. The interface between GaN and g-C_3_N_4_ induces a reorganization of electron distribution. Electrons migrate from GaN towards g-C_3_N_4_, resulting in the establishment of a built-in electric field at the interface. The inherent electric field effectively facilitates the spatial separation of photogenerated electrons and holes, thereby significantly mitigating their recombination probability. Consequently, a higher proportion of photogenerated electrons and holes can be involved in photocatalytic reactions, thus substantially advancing the photocatalytic efficiency of the catalyst [48].

### 3.2. Photocatalytic Performance

**Figure 9:**
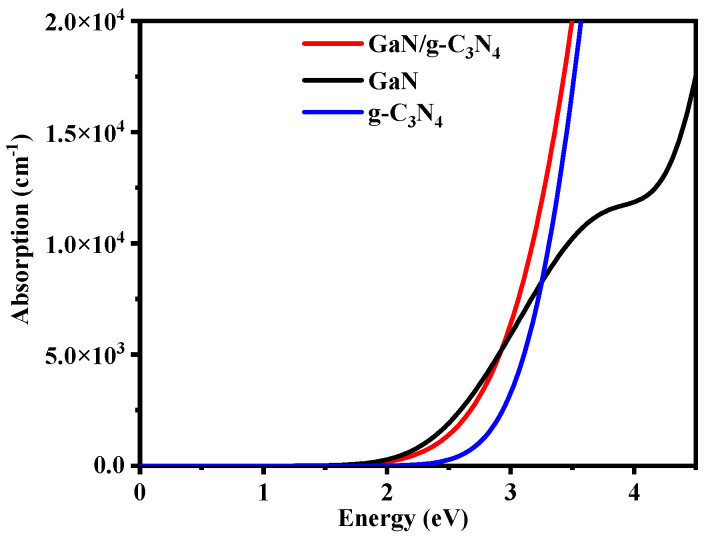
Absorption spectra of monolayer GaN, monolayer g-C_3_N_4_, and GaN/g-C_3_N_4_.

The optical absorption coefficient of GaN/g-C_3_N_4_ exhibits a remarkable enhancement within the visible light range, as illustrated in Figure 9. In comparison to monolayer g-C_3_N_4_, GaN/g-C_3_N_4_ demonstrates a significant red shift towards lower energy levels, indicating an augmented light-responsive capacity. These findings suggest that the incorporation of GaN into g-C_3_N_4_ can significantly enhance the optical absorption characteristics of this system.

**Figure 10:**
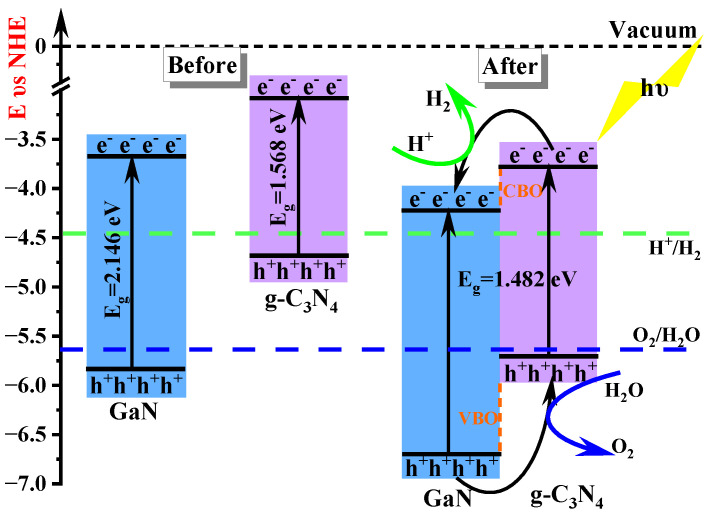
Band edge positions of GaN, g-C_3_N_4_, and GaN/g-C_3_N_4_ heterojunction for water oxidation and reduction. In the diagram, the green dashed line represents the energy level of H^+^/H_2_, while the blue dashed line corresponds to the energy level of O_2_/H_2_O.

The CBM and VBM of single-layer GaN are −3.674 eV and −5.811 eV, respectively, as illustrated in Figure 10. Similarly, the CBM and VBM of single-layer g-C_3_N_4_ are −3.084 eV and −4.649 eV, respectively. The relative positions of single-layer GaN and g-C_3_N_4_ in the band edge alignment diagram (Figure 10) are consistent with the experimental measurements in Figure 1. During the formation of a heterojunction, electron redistribution occurs at the interface between a single-layer GaN and g-C_3_N_4_, resulting in downward movement of their energy bands relative to the vacuum level. However, it is important to note that the CBM and VBM of g-C_3_N_4_ still remain at higher energy levels compared to those of GaN, leading to the formation of a staggered band structure known as type II. Therefore, our calculation results indicate that GaN/g-C_3_N_4_ is a type II heterojunction.

### 3.3. External Electric Field

**Figure 11:**
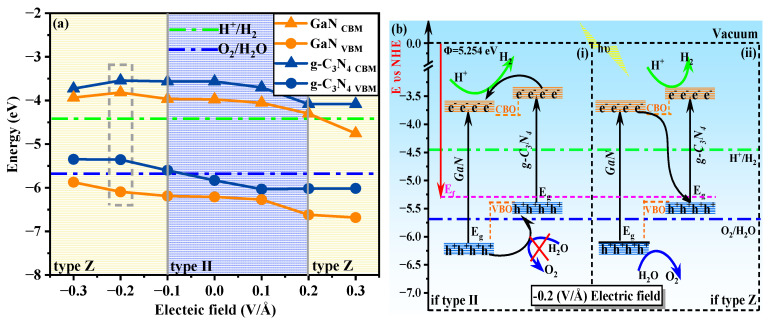
(**a**) Diagram illustrating the variation in band edge positions of GaN/g-C_3_N_4_ under the influence of sequential external electric fields for water oxidation and reduction; (**b**) Schematic representation illustrating the photocatalytic electron migration mechanism following heterojunction band edge alignment under an applied electric field intensity of −0.2 V/Å. The orange rectangle represents CB, and the blue rectangle represents VB. **b(i)** shows the assumed electron migration pathway of GaN/g-C_3_N_4_ as Type II, while **b(ii)** shows the assumed electron migration pathway of GaN/g-C_3_N_4_ as Type Z. The vacuum energy level is defined as zero eV, with the green dashed line representing oxidation potential and the blue dashed line representing reduction potential.

Extensive research has demonstrated that the bandgap and properties of heterojunction structures can be modulated by an external electric field. Therefore, in this study, we introduce an external electric field perpendicular to the planar surface of GaN/g-C_3_N_4_ along the c-direction. Figure 11a shows the variation in band edge positions of GaN/g-C_3_N_4_ under the electric field intensity range from −0.3 V/Å to 0.3 V/Å for water oxidation and reduction, wherein the valence band maximum (VBM) and conduction band minimum (CBM) of GaN and g-C_3_N_4_ are determined using formulae [49], which are expressed as follows: E_VBM_ = χ − E_elec_ + 0.5 E_g_, E_CBM_ = E_VBM_ − E_g_, wherein E_elec_ represents a constant value relative to the H electrode (E_elec_ = 4.5 eV), E_g_ denotes the bandgap of the system, and χ signifies the average electronegativity of the constituent atoms within the system [50]. According to the settlement outcome of the equations, the application of an electric field ranging from −0.1 V/Å to 0.2 V/Å sustains a higher CBM level for g-C_3_N_4_ compared to GaN while simultaneously maintaining a lower VBM. The aforementioned observation suggests that GaN/g-C_3_N_4_ possesses a staggered band structure. Furthermore, the CBM is positioned higher than the potential of H^+^/H_2_, while the VBM is situated lower than the potential of O_2_/H_2_O. The above-mentioned characteristics suggest that GaN/g-C_3_N_4_ can be classified as a type II heterojunction. When the electric field intensity falls below −0.1 V/Å or exceeds 0.2 V/Å, GaN/g-C_3_N_4_ still has a staggered band structure. However, the CBM and VBM of GaN/g-C_3_N_4_ are not fully straddled above and below the redox potential. Therefore, under electric field intensity, E ≤ −0.1 V/Å or E > 0.2 V/Å, GaN/g-C_3_N_4_ is not a type II heterojunction, but a Z-scheme heterojunction.

Subsequently, we elucidate the intrinsic mechanism of this Z-type heterojunction by considering GaN/g-C_3_N_4_ under a specific applied electric field strength (E = −0.2 V/A) as an illustrative example. Based on the aforementioned analysis, it is evident that the GaN/g-C_3_N_4_ structure exhibits a bandgap spanning characteristic, as illustrated in Figure 11b(i), suggesting its potential as a type II heterojunction [51]. However, the VBM of g-C_3_N_4_ is over the position of the water oxidation potential, thereby impeding hole transfer from the VBM of GaN to g-C_3_N_4_ and rendering it incapable of meeting the conditions required for water decomposition. This observation result negates the rationality of considering g-C_3_N_4_ as a type II heterojunction. Therefore, as illustrated in Figure 11b(ii), the electron transfer path occurs from the CBM of GaN with a lower reduced capacity to the VBM of g-C_3_N_4_ with a lower oxidized capacity, guided along a specific “Z”-type pathway, facilitating carrier recombination. Meanwhile, the electrons at the CBM of g-C_3_N_4_ with a higher reduced capacity and holes at the VBM of GaN with a higher oxidized capacity are selectively retained, facilitating effective spatial segregation of redox sites and improving the reactivity of the heterojunctions in the photocatalytic reaction. This new electron migration mechanism is termed as a direct Z-scheme [52]. According to this methodology, Z-scheme heterojunctions are formed in all GaN/g-C_3_N_4_ systems under specific electric fields (E ≤ −0.1 V/Å, E > 0.2 V/Å), as illustrated in Figure 11a.

The potential energy difference values (ΔPs) between the CBM of g-C_3_N_4_ and VBM of GaN were calculated based on the data presented in Figure 11a. The corresponding results are summarized in Table 4. It is observed from the table that the Z-scheme systems exhibit a higher ΔP compared to type II systems. The Z-type material exhibits a higher potential for photo-generated electrons compared to type II materials, thereby enhancing its reduction ability. Simultaneously, the Z-type material demonstrates a lower potential for holes in comparison to type II materials, leading to an augmented oxidation ability. ΔP serves as the underlying rationale for the enhanced separation of photogenerated carriers and redox capacity exhibited by Z-type heterojunctions when compared to type II heterojunctions.

**Figure 12:**
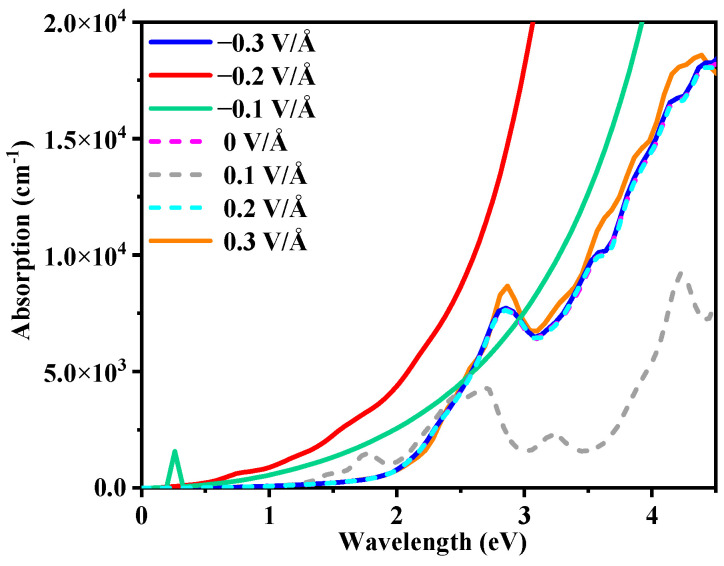
The absorption spectrum of GaN/g-C_3_N_4_ heterojunctions under the influence of different external electric field, with solid lines representing z-scheme heterojunctions and dashed lines representing type II heterojunctions.

The Z-type heterojunctions, as illustrated in Figure 12, exhibit significant redshifts compared to the type II heterojunctions. The redshift and absorption coefficients of Z-type heterojunction are notably enhanced at external electric field intensities of 0.2 V/Å and 0.1 V/Å, respectively. This observation aligns with the preamble comprehensive discussion on the Z-scheme GaN/g-C_3_N_4_, where its superior oxidation–reduction capacity has emerged as a pivotal factor in enhancing light absorption and improving photocatalytic activity.

## 4. Materials and Methods

The Vienna Ab initio Simulation Package (VASP 6.3) [53] based on density functional theory (DFT) [54] was employed in this study to investigate GaN/g-C_3_N_4_. Specifically, we selected the Perdew–Burke–Ernzerhof (PBE) exchange-correlation functional coupled with the generalized gradient approximation (GGA) [55] for our calculations. To accurately account for dispersion interactions, both the TS (Tkatchenko-Scheffler) [56] and DFT-D3 [57] methodologies were utilized. Notably, a plane wave cutoff energy of 500 eV was set, and a Monkhorst-Pack [58] scheme with a K-point grid of 3 × 3 × 1 was employed (Figure S1). Additionally, the self-consistent field (SCF) of 2 × 10^−6^ eV/atom was chosen to ensure the result precision.

## 5. Conclusions

First principle analysis was employed to investigate the electronic structure and optical properties of GaN/g-C_3_N_4_ heterojunctions under external electric fields in this study. The calculations revealed that GaN/g-C_3_N_4_ exhibits a lower lattice mismatch. AIMD simulations demonstrated minimal total energy fluctuations at 300 K, indicating robust thermodynamic stability and structural integrity. The GaN/g-C_3_N_4_ heterojunction displays a direct bandgap of 1.842 eV and exhibits a type II heterojunction configuration. The electron migration occurs from the GaN layer to the g-C_3_N_4_ layer, leading to the establishment of an intrinsic electric field directed towards the GaN layer. When the strength of the applied external electric field falls below 0.1 V/Å or exceeds 0.2 V/Å, the band structure of GaN/g-C_3_N_4_ can transition from a type II heterojunction to a Z-type heterojunction. Compared to type II materials, the Z-type material exhibits a higher potential for photo-generated electrons, which enhances its reduction ability. Additionally, it demonstrates a lower potential for holes in comparison to type II materials, resulting in an augmented oxidation ability. At external electric field intensities of 0.2 V/Å and 0.1 V/Å, respectively, the redshift and absorption coefficients of the Z-type heterojunction undergo significant enhancements. In conclusion, a precise manipulation of the external electric field can profoundly influence and optimize both band structure alignment and photocatalytic performance in these heterojunctions.

## Figures and Tables

**Table 1:** Hydrogen production rate (H), band gap (E_g_), and band structure type in g-C_3_N_4_-based heterojunctions.

Photocatalysts	H (μmol g^−1^ h^−1^)	E_g_ (eV)	Band Structure Type
Layer g-C_3_N_4_ [19]	2.82	2.70	/
g-C_3_N_4_/SiOC [20]	1020	2.64	Type II
g-C_3_N_4_/WS_2_ [21]	599.7	2.30	Type II
g-C_3_N_4_/CeO_2_ [22]	229.75	2.06	Type II

Data in Table 1 were determined through experimental measurements.

**Table 2:** Total energy of GaN/g-C_3_N_4_ heterojunctions obtained for three stacking methods using TS and Grimme dispersion correction methods.

Methods	Model I (eV)	Model II (eV)	Model III (eV)
TS	−28,134.0639	−28,134.0486	−28,134.0806
Grimine	−28,134.0480	−28,134.0237	−28,134.0534

**Table 3:** Effective masses of single-layer GaN, single-layer g-C_3_N_4,_ and GaN/g-C_3_N_4_.

System	m_e_*	m_h_*	D (m_e_*/m_h_*)
GaN	0.56	0.55	0.98
g-C_3_N_4_	0.58	0.62	1.07
GaN/g-C_3_N_4_	0.67	1.29	1.93

**Table 4:** Potential difference across GaN/g-C_3_N_4_ under varying electric fields.

Electric Field (V/Å)	−0.3	−0.2	0.3	−0.1	0	0.1	0.2
Type	Z	Z	Z	Z	II	II	II
ΔP (eV)	2.544	2.550	2.620	2.525	2.245	2.369	2.335

## Data Availability

The original contributions presented in this study are included in the article, and further inquiries can be directed to the corresponding authors.

## Supplementary Materials

The following supporting information can be downloaded at: https://www.mdpi.com/article/10.3390/molecules29225355/s1, Figure S1: Convergence test for supercell geometry optimization (a) Cutoff energy (b) K-point.
