# Supplementary material for: First Principle Study on the Z-Type Characteristic Modulation of GaN/g-C3N4 Heterojunction
Source: Molecules. 2024 Nov 14;29(22):5355. doi: 10.3390/molecules29225355 (PMC11596736; doi:10.3390/molecules29225355)
Supplement: Supplementary file 1 [file molecules-29-05355-s001.zip › molecules-3265531-supplementary.pdf]

Supplementary Material

First Principle Study on the Z-Type Characteristic Modulation of GaN/g-C_3N_4 Heterojunction

Meng-Yao Dai [1], Xu-Cai Zhao [1], Bo-Cheng Lei [1], Yi-Neng Huang [1,2], Li-Li Zhang [1,2,*], Hai Guo [3,*] and Hua-Gui Wang [1]

[1] Xinjiang Laboratory of Phase Transitions and Microstructures in Condensed Matter Physics, College of Physical Science and Technology, Yili Normal University, Yining 835000, China; dmy153097@sina.com (M.-Y.D.); zxc85619876@sina.com (X.-C.Z.); lbc0428@sina.com (B.-C.L.); ynhuang@nju.edu.cn (Y.-N.H.); suyi2046@sohu.com (H.-G.W.)

[2] National Laboratory of Solid State Microstructures, School of Physics, Nanjing University, Nanjing 210093, China

[3] Department of Physics, Zhejiang Normal University, Jinhua 321004, China

* Correspondence: suyi2046@sina.com (L.-L.Z.); ghh@zjnu.cn (H.G.)

The calculation program employed in this study is the Vienna Ab-initio Simulation Package (VASP) based on density functional theory (DFT) is employed in this study to investigate GaN/g-C_3N_4. To make the calculated results closer to the experimental values, we performed a convergence test for the cutoff energy before conducting geometric optimization. As shown in Fig. S1(a), the results indicate that energy gradually stabilizes when cutoff energy is set to 500 eV. Therefore, we selected 500 eV as the cutoff energy for the system. Additionally, we performed further convergence tests on the K-point and found that the total energy reaches its minimum value when using a 3×3×1 K-point. Consequently, we set both plane wave cutoff energy and Monkhorst-Pack scheme with a K-point grid of 3×3×1.

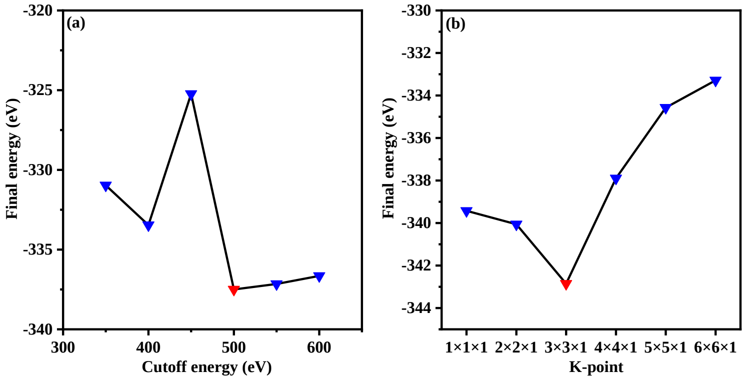

Figure S1. Convergence test for supercell geometry optimization (**a**) Cutoff energy (**b**) K-point.
